# Urinary Angiotensinogen Could Be a Prognostic Marker of Renoprotective Effects of Alogliptin in Patients with Type 2 Diabetes

**DOI:** 10.1155/2015/517472

**Published:** 2015-08-25

**Authors:** Tomoko Mizushige, Hiroyuki Kobori, Yoko Nishijima, Yuichiro Yano, Koji Sakata, Manabu Hayakawa, Akira Nishiyama

**Affiliations:** ^1^Kagawa University School of Medicine, Kagawa 761-0793, Japan; ^2^Jichi Medical University School of Medicine, Tochigi, Japan; ^3^Tulane University Health Sciences Center, New Orleans, LA, USA; ^4^Miyazaki University School of Medicine, Miyazaki, Japan

## Abstract

*Background*. The aims of this study were (1) to examine the renoprotective effects of alogliptin and (2) to establish urinary angiotensinogen (AGT) as a prognostic marker of renoprotective effects of alogliptin in patients with type 2 diabetes (T2D).* Methods*. In 43 patients with T2D (18 women, 66.1 ± 1.71 years), 25 mg/day of alogliptin was added to the traditional hypoglycemic agents and/or nondrug treatments. Urinary concentrations of albumin (Alb) and AGT, normalized by urinary concentrations of creatinine (Cr) (UAlbCR and UAGTCR, respectively), were measured before and after the 12-week alogliptin treatment.* Results*. Alogliptin treatment tended to decrease UAlbCR (99.6 ± 26.8 versus 114.6 ± 36.0 mg/g Cr, *P* = 0.198). Based on % change in UAlbCR, patients were divided into two groups, responders (<−25%) and nonresponders (≥−25%), and a logistic analysis of UAGTCR before treatment showed cutoff value of 20.8 *µ*g/g Cr. When all patients were redivided into two groups, those with higher values of UAGTCR before the treatment (Group H, *n* = 20) and those with lower values (Group L), Group H showed significantly decreased UAlbCR in response to alogliptin (−14.6 ± 8.6 versus +22.8 ± 16.8%, *P* = 0.033).* Conclusion*. Urinary AGT could be a prognostic marker of renoprotective effects of alogliptin in patients with T2D.

## 1. Introduction

Diabetic nephropathy is one of the greatest primary diseases necessitating hemodialysis in patients with end-stage renal disease. It is very important to control the development of symptoms and progress of nephropathy in the medical treatment of diabetes. However, the detailed mechanisms of the development and progression of diabetic nephropathy are still unknown.

There are many reports indicating that the increase of intrarenal angiotensinogen (AGT) and the activation of the renin-angiotensin system (RAS) are involved in diabetic nephropathy [1–6].

On the other hand, it is reported that medicines associated with incretin, such as dipeptidyl peptidase- (DPP-) 4 inhibitors and glucagon like peptide-1, have a renoprotective effect in addition to improving glycemic control [7–10]. However, there are few reports about the relationship between DPP-4 inhibitors and RAS in the kidneys. It has been reported that urinary AGT is a useful marker of intrarenal RAS activity [11] in patients with chronic kidney disease [12, 13] as well as in patients with diabetes [14–17]. Therefore, this study was performed to demonstrate that urinary AGT may serve as a prognostic marker of the renoprotective effect of DPP-4 inhibitors.

## 2. Materials and Methods

### 2.1. Patients and Protocols

This experimental protocol was approved by the institutional review board of Social Insurance Miyazaki Konan Hospital (Miyazaki, Japan). Patients with type 2 diabetes (T2D) were recruited from Miyazaki University affiliated hospitals from August 2011 to June 2012, and written informed consents were obtained. T2D was defined as fasting blood glucose ≥126 mg/dL, glycated hemoglobin (HbA1c) ≥6.5%, according to the guidelines of the American Diabetes Association, or receiving treatments with oral hypoglycemic agents. Patients whose HbA1c levels were ≥6.1% for at least 3 months, in spite of nondrug treatments (i.e., exercise, diet, and lifestyle modification) or medications with a stable dose of oral hypoglycemic agents (except DPP-4 inhibitors), were included. Patients (1) with administration of insulin; (2) with hepatic or renal impairment (aspartate aminotransferase or alanine aminotransferase ≥2.5 × upper limit of normal or serum creatinine (Cr) ≥2 mg/dL); (3) with cardiovascular disease within 6 months (i.e., myocardial infarction or stroke); (4) taking a moderate or high dose of glimepiride (i.e., >3 mg/day); (5) and taking sulfonylureas other than glimepiride were excluded. Other antihyperglycemic and antihypertensive medications were not changed during this study.

### 2.2. Measurements

The patients took 25 mg alogliptin once daily for 12 weeks, and serum HbA1c, serum Cr, urinary albumin (Alb), urinary AGT, and urinary Cr were measured, at baseline and after 12 weeks.

HbA1c concentration, determined using a latex agglutination immunoassay, was estimated as a National Glycohemoglobin Standardization Program (NGSP) equivalent value calculated with the following formula: HbAlc (NGSP, %) = HbAlc (Japan Diabetes Society, %) + 0.4. To estimate renal function, the estimated glomerular filtration rate (eGFR) derived using the following equation was used: eGFR (mL/min/1.73 m^2^) = 194 × age (years)^−0.287^  ×  serum Cr (mg/dL)^−1.094^ (if women × 0.739) [18]. The urinary concentrations of Alb were measured using an immune-turbidity kit (AutoWako Microalbumin; Wako Pure Chemical Industries, Ltd., Osaka, Japan) and expressed as a Cr ratio (UAlbCR, mg/g Cr) by spot urine. The intra-assay and interassay coefficients of the Alb measurements were all <10%. Urinary AGT was measured using the Human Total AGT ELISA Kit (Immuno-Biological Laboratories Co. Ltd., Takasaki, Japan) according to the manufacturer's instructions as previously described [19] by spot urine and also normalized by urinary concentrations of Cr (UAGTCR, *μ*g/g Cr). The intra-assay and interassay coefficients of the AGT measurements were all <10% [19].

### 2.3. Statistical Analysis

All statistical analyses were performed with JMP software version 10 (SAS Institute Inc., Tokyo, Japan). Age, body mass index (BMI), and treatment duration of T2D were expressed as means ± standard error (SE). UAlbCR, UAGTCR, HbA1c, eGFR, systolic blood pressure (SBP), and diastolic blood pressure (DBP) before and after treatment by alogliptin were expressed as means ± SE and were compared using a paired* t*-test. Logistic analysis of UAGTCR before treatment was conducted whether UAlbCR decreased more than 25% after alogliptin treatment or not. We developed a receiver operator characteristic (ROC) curve; the area under the curve (AUC) was calculated and the optimal cutoff value was determined.

Based on this cutoff value of UAGTCR before the treatment, we divided all patients into 2 groups. We compared % change in UAlbCR and ΔUAlbCR between the 2 groups using an unpaired* t*-test. *P* value < 0.05 was defined as statistically significant.

## 3. Results

### 3.1. Baseline Patients Profiles

43 patients (18 women and 25 men, 66.1 ± 1.71 years) were assigned to treatment. The patients' clinical characteristics and laboratory data before and after treatment by alogliptin are summarized in Tables 1 and 2, respectively. There are significant changes in HbA1c, but there is no significant change in other parameters before and after the treatment.

### 3.2. UAlbCR before and after the Treatments by Alogliptin

**Figure 1:**
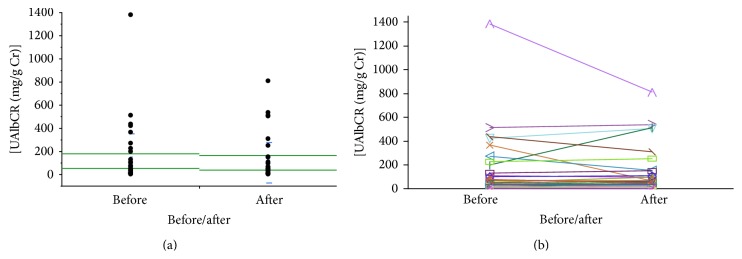
(a) Urinary concentrations of Alb normalized by urinary concentrations of creatinine (UAlbCR) before and 12-week after treatment with alogliptin. Alogliptin treatment tended to decrease UAlbCR (99.6 ± 26.8 versus 114.6 ± 36.0 mg/g Cr, *P* = 0.1976). (b) UAlbCR of each participant before and 12-week after treatment with alogliptin.

The alogliptin treatment tended to decrease UAlbCR (99.6 ± 26.8 versus 114.6 ± 36.0 mg/g Cr) with no statistically significant changes (*P* = 0.1976, Figure 1(a)). We also showed UAlbCR of each participant before and after treatment by alogliptin in Figure 1(b).

### 3.3. Logistic Analysis of UAGTCR before the Treatments

**Figure 2:**
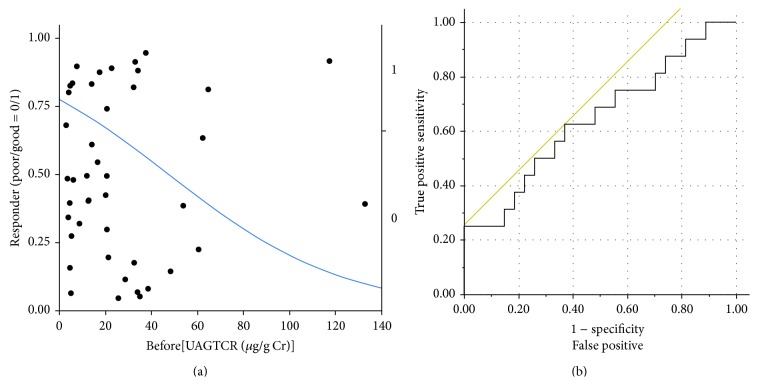
Logistic analysis of urinary concentrations of angiotensinogen normalized by urinary concentrations of creatinine (UAGTCR) before treatment. Good responders to the alogliptin treatment were defined in terms of % change in urinary concentrations of Alb normalized by urinary concentrations of creatinine less than −25% after the 12-week treatment, and a logistic analysis of UAGTCR before treatment showed the area under the curve as 0.644. When we set the cutoff value of UAGTCR as 20.8 *μ*g/g Cr, the maximum specificity (17/27 = 63.0%) and sensitivity (10/16 = 62.5%) were obtained (Youden index = 0.255).

Patients were divided into two groups: those for whom UAlbCR decreased less than 25% and those for whom it decreased more than 25%. A logistic analysis of UAGTCR before treatment showed AUC as 0.644. When we set the cutoff value of UAGTCR as 20.8 *μ*g/g Cr, the maximum specificity (17/27 = 63.0%) and sensitivity (10/16 = 62.5%) were obtained (Youden index = 0.255, Figures 2(a) and 2(b)).

### 3.4. Clinical Characteristics and Baseline Laboratory Data Partitioned by the Cutoff Value of UAGTCR before the Treatments

**Figure 3:**
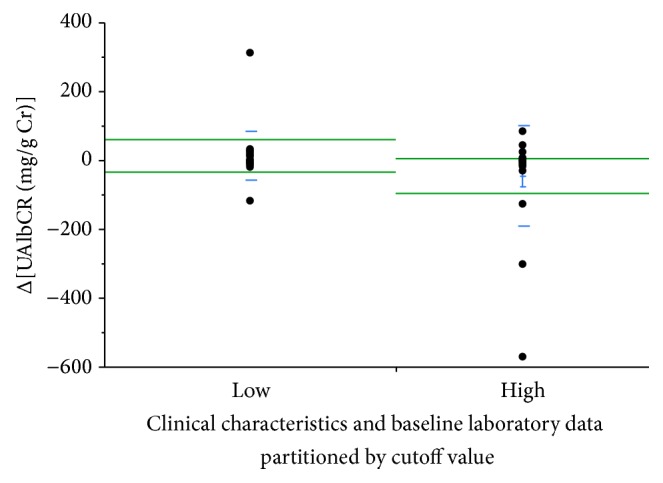
ΔUrinary concentrations of Alb normalized by urinary concentrations of creatinine (UAlbCR) defined by the cutoff value of urinary concentrations of angiotensinogen normalized by urinary concentrations of creatinine (UAGTCR) before treatment. When all patients were redivided into two groups, those with higher UAGTCR levels before treatment (Group H, *n* = 20) and those with lower levels (Group L), ΔUAlbCR was significantly lower in Group H than in Group L (−46.3 ± 32.5 versus +12.2 ± 14.9 mg/g Cr, *P* = 0.0474).

Based on this cutoff value of UAGTCR (i.e., ≥20.8 *μ*g/g Cr or <20.8 *μ*g/g Cr), we divided all patients into 2 groups: higher (Group H, *n* = 20) and lower (Group L) values of UAGTCR at baseline. Clinical characteristics (Table 3) and baseline laboratory data (gender, age, BMI, treatment duration of T2D, HbA1c (NGSP), eGFR, SBP, DBP, and medications) (Table 4) were not significantly different between Group H and Group L. However, ΔUAlbCR was significantly lower in Group H than in Group L (−46.3 ± 32.5 versus +12.2 ± 14.9 mg/g Cr, *P* = 0.0474, Figure 3).

**Figure 4:**
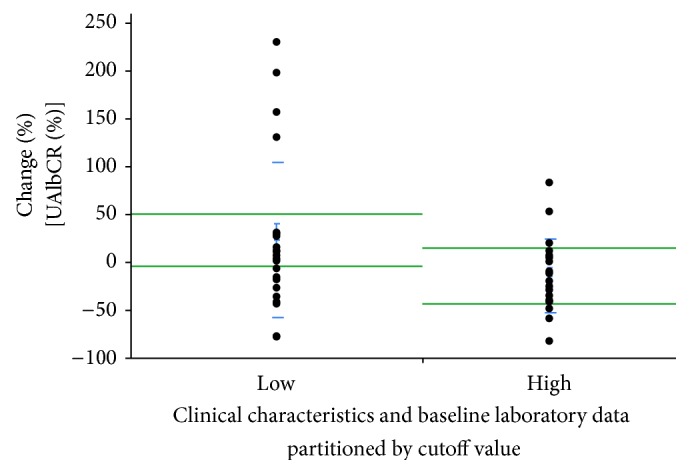
% change in urinary concentrations of Alb normalized by urinary concentrations of creatinine (UAlbCR) defined by the cutoff value of urinary concentrations of angiotensinogen normalized by urinary concentrations of creatinine (UAGTCR) before treatment. When all patients were redivided into two groups, those with higher UAGTCR levels before treatment (Group H, *n* = 20) and those with lower levels (Group L), % change in UAlbCR was significantly lower in Group H than in Group L (−14.6 ± 8.6% versus +22.8 ± 16.8%, *P* = 0.0327).

In addition, % change in UAlbCR was significantly lower in Group H than in Group L (−14.6 ± 8.6 versus +22.8 ± 16.8%, *P* = 0.0327, Figure 4).

## 4. Discussion

Recently, there has been increased emphasis on the role of the local/tissue RAS in specific tissues in organ injury. The importance of the tissue RAS was demonstrated in the brain, heart, adrenal glands, vasculature, and kidneys [1]. In the kidneys, there are AGT [20], renin [21, 22], and angiotensin-converting enzyme [23] in the proximal and distal tubular cells, and they were converted to angiotensin II. Once angiotensin II concentration increases within the kidneys, AGT, which is the substrate of angiotensin II, will be increased further [20]. In terms of the origin of the intrarenal AGT and angiotensin II, Matsusaka et al. reported that liver AGT is the primary source of renal angiotensin II [24, 25]. Meanwhile, Nakano et al. suggest that the vast majority of urinary AGT originates from the tubules rather than glomerular filtration [26]. These papers seem to provide conflicting findings. Further investigations are still needed to address this important issue.

The intrarenal RAS is involved in the development and progression of renal damage [11–13]. In rat glomerular cells, increasing angiotensin II causes an increase of transforming growth factor *β*1, and causes renal damage by hypertrophy and fibrosis [27, 28]. Activation of the intrarenal RAS was also involved in diabetic nephropathy, and urinary AGT was increased in T2D model rat [3].

The underlying mechanisms of the development and progression of diabetic nephropathy are still under investigation. Diabetic nephropathy is associated with the increased reactive oxygen species (ROS) and involves various mechanisms, including hyperglycemia, activation of the intrarenal RAS, and high blood pressure. Hyperglycemia induces acyl glycerol and activates protein kinase C. Glomerular injury is caused by the generation of ROS or the peroxidation of lipids [29]. Hyperglycemia produces ROS as a result of the self-oxidation of glucose, metabolism, or formation of advanced glycation end-product [30].

In terms of activation of the intrarenal RAS, it has been demonstrated that ROS and intrarenal AGT levels increase in diabetic rats [4, 5] and humans [6] before generating renal damage. Moreover, Ogawa et al. [31] demonstrated that angiotensin II receptor blocker (ARB) treatment reduces urinary Alb levels at the stage of microalbuminuria underlying diabetic nephropathy when the urinary oxidative stress marker and AGT are high. The activation of the RAS is thought to be strongly associated with the increase in production of ROS. In the stage of microalbuminuria, urinary Alb is decreased along with decreasing production of intrarenal ROS and controlling the activity of the RAS by ARB treatments. There is a complex relationship between Alb reabsorption and the production of ROS and AGT. Unfortunately, however, the samples were not remaining anymore and it is impossible for us to measure urinary markers of oxidative stress in this study.

This study focused on the effects of a DPP-4 inhibitor on urinary AGT in patients with diabetes. Even though ARB may affect AGT synthesis [3] and urinary AGT [12, 15], the effects of ARB on urinary AGT in patients with diabetes are beyond the scope of this study, and we need another study to address this issue. However, the important point is that patients on ARB medication were 12 (60.0%) in Group H and 11 (47.8%) in Group L, and there are no significant differences between Group H and Group L (*P* = 0.54, Table 3). Therefore, this issue did not affect the results so much in this study.

It is assumed that the increase in intrarenal AGT and ROS formation underlying T2D [15, 31] is associated with the onset of diabetic nephropathy [1, 2, 14]. It has been reported that DPP-4 inhibitor medications improved the marker of oxidative stress in the kidney and had a renoprotective effect [7–10]. These mechanisms may be achieved by the direct action of DPP-4 inhibitors or by the indirect action of DPP-4 inhibitors through the improvement of hyperglycemia [7–10].

In the RENAAL study [32], it is reported that losartan, an ARB, reduced the risk of end-stage renal disease by 28% in patients with T2D and nephropathy (incidence: 147/751 = 19.6% in the losartan group versus 194/762 = 25.5% in the placebo group). This investigator-initiated, multinational, double-blind, randomized, placebo-controlled study has a significant impact demonstrating that ARB exerts renoprotective effect in patients with T2D. However, this study also suggested that 19.6% of patients with T2D undergo end-stage renal disease even though they are treated with losartan. This indicated that there are responders and nonresponders for losartan. If we are able to predict the response of losartan by some biomarkers before patients receive the treatment by losartan, we may be able to increase the responders to losartan. This may increase the clinical impact and reduce the medical cost on patients with T2D. In this regard, we believe that our approach is valid and makes an important step to lead to the tailor-maid medicine. In this study, responders who showed a decreased UAlbCR by 25% or more are 16 patients, and nonresponders are 27 patients. Therefore, 37.2% (16/43) of all patients in this study are responders. Based on the cutoff value of UAGTCR before the treatment, we divided all patients into 2 groups: higher (Group H, *n* = 20) and lower (Group L) values of UAGTCR at baseline. In group H, 10 patients (50%) are responders. In this way, we may be able to increase the number of responders to a DPP-4 inhibitor. If we are able to predict the renoprotective effect of a DPP-4 inhibitor by some biomarkers before the treatment, this has a clinical impact and a possibility to reduce the medical costs on patients with T2D. UAGTCR is a useful biomarker to predict the renoprotective effect of a DPP-4 inhibitor, as demonstrated in this study.

We chose UAGTCR as biomarker not UAlbCR. Urinary AGT shows a positive correlation with urinary Alb in this study as well as in other studies. However, an increase in urinary AGT appears earlier than an increase in urinary Alb in diabetes. Others [33] and we [16] previously reported that an increase in urinary AGT is observed in normoalbuminuric children with type 1 diabetes. In addition, we reported that an increase in urinary AGT precedes an increase in urinary Alb in experimental type 1 diabetes [34]. These data suggest that urinary AGT and urinary Alb were not identical.

In this study, not only the amount of change but also the rate of change in urinary Alb displayed a larger drop in the higher urinary AGT group. An amount of change will tend to be larger when one starts with higher levels. However, a rate of change will tend to be larger when one starts with lower levels. Because both the amount of change and the rate of change in urinary Alb significantly decreased in the higher urinary AGT group, these results in this study were very important.

In this study, UAlbCR tended to fall after treatment with DPP-4 inhibitor and possibly exerted a renoprotective effect. However, the difference was not statistically significant, possibly due to the small sample size. In this study, there is also a restriction of the amount of samples. Therefore, the data addressing the underlying mechanism cannot be measured in this study. Further studies will be required to address this issue. We are now planning a multicenter randomized prospective study on urinary AGT as a prognostic marker of renoprotective effects of DPP-4 inhibitors in patients with T2D.

In conclusion, treatments of alogliptin in patients with T2D may protect kidney function in some patients. Urinary AGT could be a prognostic marker of renoprotective effects of alogliptin in patients with T2D.

## Figures and Tables

**Table 1:** Patient profiles.

*N*	43
Men/women	25/18
Age (years)	66.1 ± 1.71
BMI (kg/m^2^)	24.8 ± 0.5
Treatment duration of T2D (years)	7.1 ± 1.18
Medications	
ARB	23 (53.5%)
*α*-GI	8 (18.6%)
TZD	5 (11.6%)

ARB: angiotensin II receptor blockers, *α*-GI: *α*-glucosidase inhibitors, and TZD: thiazolidines.

**Table 2:** Laboratory data of before and after treatment by alogliptin.

	Before	After	*P* value
UAlbCR (mg/g Cr)	114.6 ± 36.0	99.6 ± 26.8	*P* = 0.198
UAGTCR (*µ*g/g Cr)	27.2 ± 4.2	29.9 ± 8.0	*P* = 0.628
HbA1c (NGSP) (%)	7.2 ± 0.1	7.0 ± 0.1	*P* = 0.005^*^
eGFR (mL/min/1.73 m^2^)	74.3 ± 3.1	72.2 ± 3.0	*P* = 0.067
Systolic blood pressure (mmHg)	140.5 ± 2.9	138.3 ± 2.9	*P* = 0.130
Diastolic blood pressure (mmHg)	77.8 ± 1.7	78.7 ± 1.6	*P* = 0.763

^*^ *P* value < 0.05.

**Table 3:** Patient profiles partitioned by the cutoff value of UAGTCR before treatments.

Group	High	Low	*P* value
*N*	20	23	
Men/women	11/9	14/9	*P* = 0.76
Age (years)	67.0 ± 2.5	65.4 ± 2.4	*P* = 0.66
BMI (kg/m^2^)	24.1 ± 0.7	25.4 ± 0.7	*P* = 0.19
Treatment duration of T2D (years)	6.9 ± 1.8	7.4 ± 1.6	*P* = 0.82
Medications			
ARB	12 (60.0%)	11 (47.8%)	*P* = 0.54
*α*-GI	4 (20.0%)	4 (17.4%)	*P* = 1.00
TZD	1 (5.00%)	4 (17.4%)	*P* = 0.35

ARB: angiotensin II receptor blockers, *α*-GI: *α*-glucosidase inhibitors, and TZD: thiazolidines.

**Table 4:** Laboratory data at the entry partitioned by the cutoff value of UAGTCR before treatments.

Group	High	Low	*P* value
HbA1c (NGSP) (%)	7.26 ± 0.15	7.23 ± 0.14	*P* = 0.89
eGFR (mL/min/1.73 m^2^)	76.6 ± 4.6	72.3 ± 4.3	*P* = 0.50
Systolic blood pressure (mmHg)	143.6 ± 4.3	137.9 ± 4.0	*P* = 0.33
Diastolic blood pressure (mmHg)	77.0 ± 2.5	78.4 ± 2.4	*P* = 0.68
